# The Gut Microbiome Signatures Discriminate Healthy From Pulmonary Tuberculosis Patients

**DOI:** 10.3389/fcimb.2019.00090

**Published:** 2019-04-03

**Authors:** Yongfei Hu, Yuqing Feng, Jiannan Wu, Fei Liu, Zhiguo Zhang, Yanan Hao, Shihao Liang, Boxing Li, Jing Li, Na Lv, Yuhui Xu, Baoli Zhu, Zhaogang Sun

**Affiliations:** ^1^State Key Laboratory of Animal Nutrition, College of Animal Science and Technology, China Agricultural University, Beijing, China; ^2^CAS Key Laboratory of Pathogenic Microbiology and Immunology, Institute of Microbiology, Chinese Academy of Sciences, Beijing, China; ^3^Translational Medicine Research Center, Beijing Chest Hospital, Capital Medical University, Beijing, China; ^4^Department of Laboratory Medicine, Tuberculosis Control Institute in Changping District, Beijing, China; ^5^Institute of Chinese Materia Medica, China Academy of Chinese Medical Sciences, Beijing, China; ^6^Collaborative Innovation Center for Diagnosis and Treatment of Infectious Diseases, The First Affiliated Hospital, Zhejiang University, Hangzhou, China; ^7^Department of Pathogenic Biology, School of Basic Medical Sciences, Southwest Medical University, Luzhou, China

**Keywords:** human gut microbiota, tuberculosis, metagenomic sequencing, microbial diversity, metabolic potential

## Abstract

Cross talk occurs between the human gut and the lung through a gut-lung axis involving the gut microbiota. However, the signatures of the human gut microbiota after active *Mycobacterium tuberculosis* infection have not been fully understood. Here, we investigated changes in the gut microbiota in tuberculosis (TB) patients by shotgun sequencing the gut microbiomes of 31 healthy controls and 46 patients. We observed a dramatic changes in gut microbiota in tuberculosis patients as reflected by significant decreases in species number and microbial diversity. The gut microbiota of TB patients were mostly featured by the striking decrease of short-chain fatty acids (SCFAs)-producingbacteria as well as associated metabolic pathways. A classification model based on the abundance of three species, *Haemophilus parainfluenzae, Roseburia inulinivorans*, and *Roseburia hominis*, performed well for discriminating between healthy and diseased patients. Additionally, the healthy and diseased states can be distinguished by SNPs in the species of *B. vulgatus*. We present a comprehensive profile of changes in the microbiota in clinical TB patients. Our findings will shed light on the design of future diagnoses and treatments for *M. tuberculosis* infections.

## Introduction

Tuberculosis (TB) is one of the most important infectious diseases, with extremely high morbidity and mortality worldwide. The World Health Organization (WHO) estimated that there were 10.4 million new TB cases and 1.4 million TB deaths worldwide in 2015 (WHO, 2016). *Mycobacterium tuberculosis* is the causative agent of TB, and although it can infect extrapulmonary organs, such as lymph nodes, bone, and the meninges, *M. tuberculosis* predominantly infects the lungs and causes pulmonary TB (Harisinghani et al., 2000).

The human body is inhabited by a tremendous number of commensal bacteria (the microbiota) that closely communicate with the human immune system and exert significant influence on human health. Various infectious and non-infectious diseases are associated with the human microbiota, particularly the gut microbiota (Young, 2017). Given the close connections between the gut microbiota and diseases of the nervous system, liver and lung, the gut microbiota is proposed to be involved in the gut-brain (Foster and Neufeld, 2013), gut-liver (Compare et al., 2012) and gut-lung (Budden et al., 2017) axes, with the latter being the most recently recognized. With respect to the gut-lung axis hypothesis, dynamic cross talk between the microbiota of the respiratory tract and the gut has been suggested. The processes are bidirectional, occurring either via the translocation of local bacteria to the other site or through the release of immunomodulatory molecules (bacteria-derived components or metabolites, such as short-chain fatty acids [SCFAs]) into the bloodstream, thus affecting systemic immunity (Marsland et al., 2015; Budden et al., 2017; Cervantes and Hong, 2017). Many studies have focused on revealing the role of this axis in the development of lung diseases, such as asthma (Abrahamsson et al., 2014), chronic obstructive pulmonary disease (COPD) (Ekbom et al., 2008), and pneumococcal and *Staphylococcus aureus* pneumonia (Gauguet et al., 2015; Schuijt et al., 2015).

To date, several efforts have been made to reveal the gut microbiota changes after *M. tuberculosis* infection using murine models or clinical samples (Dubourg et al., 2013; Winglee et al., 2014; Luo et al., 2017; Namasivayam et al., 2017; Wipperman et al., 2017). However, whether there are microbiota signatures in gut microbial composition or metabolic potentials that can discriminate healthy from *M. tuberculosis* infection has not been evaluated. Here, we characterized changes of the gut microbiota in the fecal samples of clinical pulmonary TB patients. We observed dramatic alterations in the structure and metabolic pathways of the gut microbiota in the TB patients, and showed that the gut bacterial signatures have the potentials to be used for discriminating healthy from TB patients.

## Materials and Methods

### Study Cohort

We first sequenced the metagenomes of 61 fecal samples acquired from 31 healthy controls (C group) and 30 TB patients (P group). Then another 16 patients were sequenced and used as the patient group (Test-P) in the testing dataset in the subsequent discrimination model. All the 46 subjects in the P group were newly diagnosed with active pulmonary TB prior to anti-TB treatment. Patients suffering from other serious diseases were excluded. The subjects were diagnosed with TB by assessing symptoms, including the results of acid-fast bacilli (AFB) smear microscopy, culture, the T-SPOT.TB test and a chest radiograph. Drug-susceptibility testing was performed as described previously (Zhao et al., 2012). This study was approved by local ethics committees (Beijing Chest Hospital, Capital Medical University, China), and written informed consent was obtained from all participants.

### Stool Sample Collection and DNA Extraction

Fresh stool samples from the healthy controls and the patients were collected using collection tubes containing stool DNA stabilizer provided in the PSP® Spin Stool DNA Plus Kit (Stratec Molecular, Germany), and stored at −20°C until DNA extraction. Metagenomic DNA was extracted from 200 mg of feces by using the recommended kit per the manufacturer.

### Metagenomic Sequencing and Data Processing

The DNA sequencing libraries with insert sizes of 350 bp were constructed following the manufacturer's instructions (Illumina). The libraries were then paired-end sequenced on the HiSeq 2500 Illumina sequencers. The raw sequencing data were processed using the MOCAT2 (Kultima et al., 2016) pipeline to remove low-quality reads, adapters and human DNA contamination. In brief, reads were trimmed using a length cut off of 30 and a quality cut-off of 20. Next, the trimmed reads were then screened against Illumina adapter sequences with an e-value of 0.01, and against the human genome sequence with a 90% identity cut-off. After filtering, approximately 5 GB of clean data for each sample was obtained on average. For functional profiling, genes were predicted and clustered into reference gene catalogs after the clean data was assembled. The taxonomic assignment and abundance estimation were performed with MetaPhlAn2 (Truong et al., 2015) using default parameters.

### Microbial Diversity and Enterotype Analysis

The within-sample (α) diversity of samples was calculated using the Shannon index based on the species profile. The distance between samples (β diversity) was estimated by Non-metric multidimensional scaling (NMDS) analysis based on the relative abundance of genera.

### Functional Profiling and Metabolic Pathway Analysis

Metabolic pathway analysis was performed using clean data and the program HUMAnN2 (Abubucker et al., 2012), which uses the MetaCyc Metabolic Pathway Database (Caspi et al., 2015) for annotation. MetaCyc currently contains 2526 experimentally elucidated metabolic pathways involved in both primary and secondary metabolism.

### Random Forest Model Construction and Validation

For discrimination analysis using the random forest package in R, a random forest classifier was trained on the species abundance profile of the C (*n* = 31) and P (*n* = 30) groups. Five repeats of 10-fold cross-validation were performed to select the optimal number of species used for the model. The random forest variable importance by mean decrease in accuracy and in gini was calculated. The first three species with the lowest cross-validation errors were used for the predictive model construction, in which the receiving operational curve (ROC) was analyzed and the area under the ROC (AUC) was calculated. To evaluate the discriminatory ability of the model, an independent dataset comprised of 16 patients (Test-P group, mentioned above) and 30 healthy controls (Test-H group) was used for validation. The 30 controls were randomly selected from the healthy control group used in a type 2 diabetes (T2D) cohort study reported previously (Qin et al., 2012). The original sequencing data from these samples were downloaded from NCBI database and then taxonomically profiled using the same procedure applied for our samples as described above.

### Metagenomic SNP Calling and Phylogenetic Analysis

The identification of metagenomic SNPs was performed using the Metagenomic Intra-species Diversity Analysis System (MIDAS) (Nayfach et al., 2016). In brief, SNP calling was carried out when species had sufficient sequencing coverage and depth. First, a local bowtie2 database containing one representative genome for each abundant species, was built. Reads in each sample were then aligned to the genome to identify SNPs and to estimate allele frequencies. The following filtering criteria were used: ≥40% of the reference genome was covered with >15× average depth; SNP sites were covered by ≥10 reads in at least 20% of samples in each group. For heterozygous SNPs present in a sample, the major allele was used for the SNP analysis. Concatenated core-genome (commonly covered genomic regions in different samples) SNPs were used for phylogenetic analysis. The maximum likelihood (ML) tree was constructed by MEGA 6 (Tamura et al., 2013) with 1000 bootstrap replicates.

### Statistics

All statistical analyses were performed using R packages. The Wilcoxon rank-sum test was used to compare continuous variables. Two-tailed Fisher's exact test was used to compare differences in SNP distributions between groups. Spearman's rank test was performed to analyze the correlation between species. When multiple hypothesis tests were performed simultaneously, *P*-values were corrected using Benjamini and Hochberg's false discovery rate (FDR) (Benjamini and Hochberg, 1995).

## Results and Discussion

### Microbial Diversity is Decreased in TB Patients

**Figure 1:**
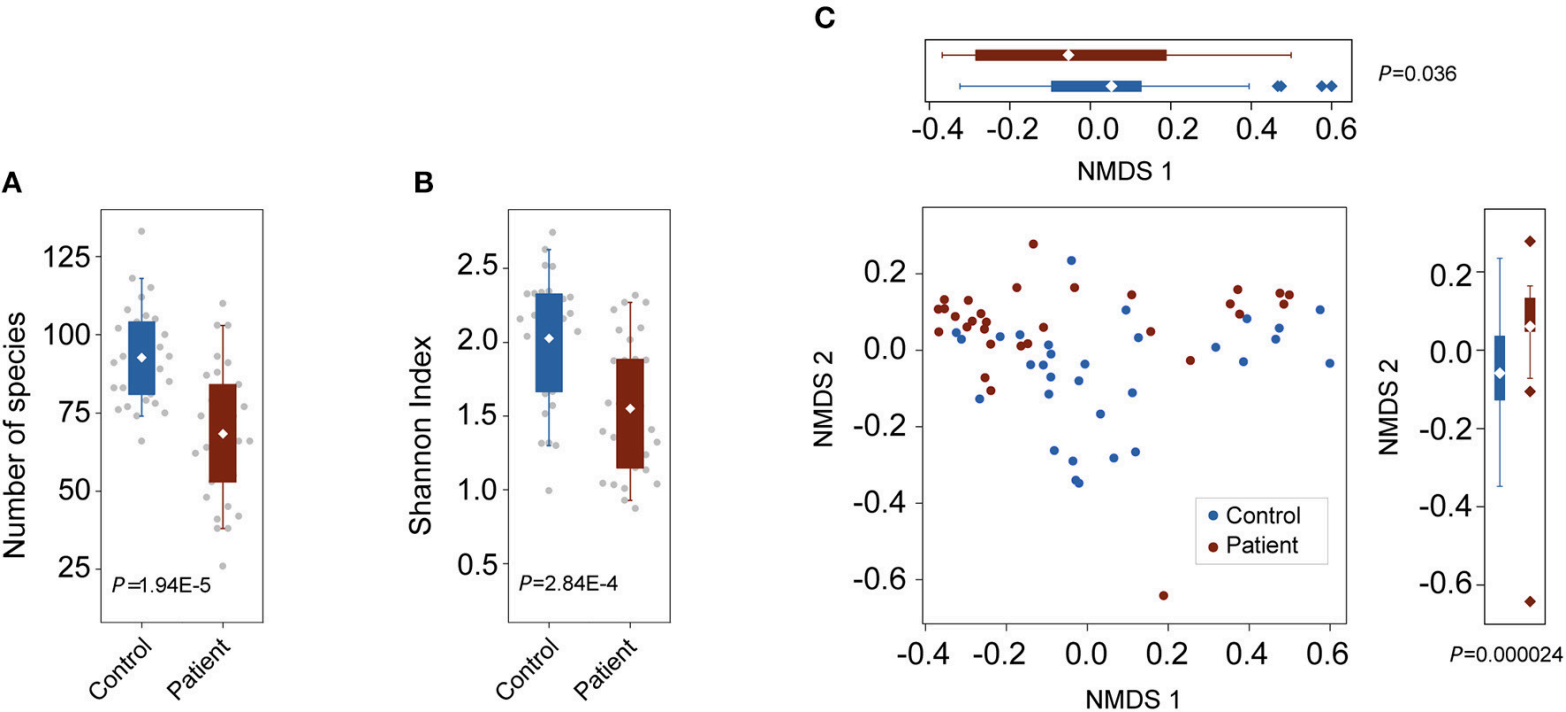
Changes in bacterial diversities in gut microbiota in TB patients compared with healthy controls. C, control; P, patients. **(A)** Comparison of the numbers of species in the two groups. *P* = 1.94E-5, Wilcoxon signed-rank test. **(B)** Comparison of α-diversity (as assessed by the Shannon index). *P* = 2.84E-4, Wilcoxon rank-sum test. **(C)** NMDS plots based on the relative abundance of genera. Values of NMDS axes 1 and 2 for three groups were box plotted on the top and the right, respectively.

To characterize alterations in the gut microbiota in TB patients, we analyzed the microbial composition and metabolic potential of the gut microbiota in 61 samples from the C (*n* = 31) and P (*n* = 30) groups (Table S1). Using MetaPhlAn2 for taxonomic profiling, we identified 11 phyla, 156 genera and 400 species across the three groups (Tables S2, S3, S4). Compared with the number of species present in the controls, the species present in the patients was significantly decreased (*P* = 1.95E-5; Wilcoxon rank-sum test; Figure 1A). Consistently, the Shannon index, indicating within-sample (α) diversity, was much lower for the P groups than that for the controls (*P* = 2.84E-4; Wilcoxon rank-sum test; Figure 1B). NMDS analysis based on genus relative abundance also revealed striking differences between the C and P groups especially at the NMDS 2 axis (Figure 1C).

### Gut Bacteria Associated With *M. tuberculosis* Infection

**Figure 2:**
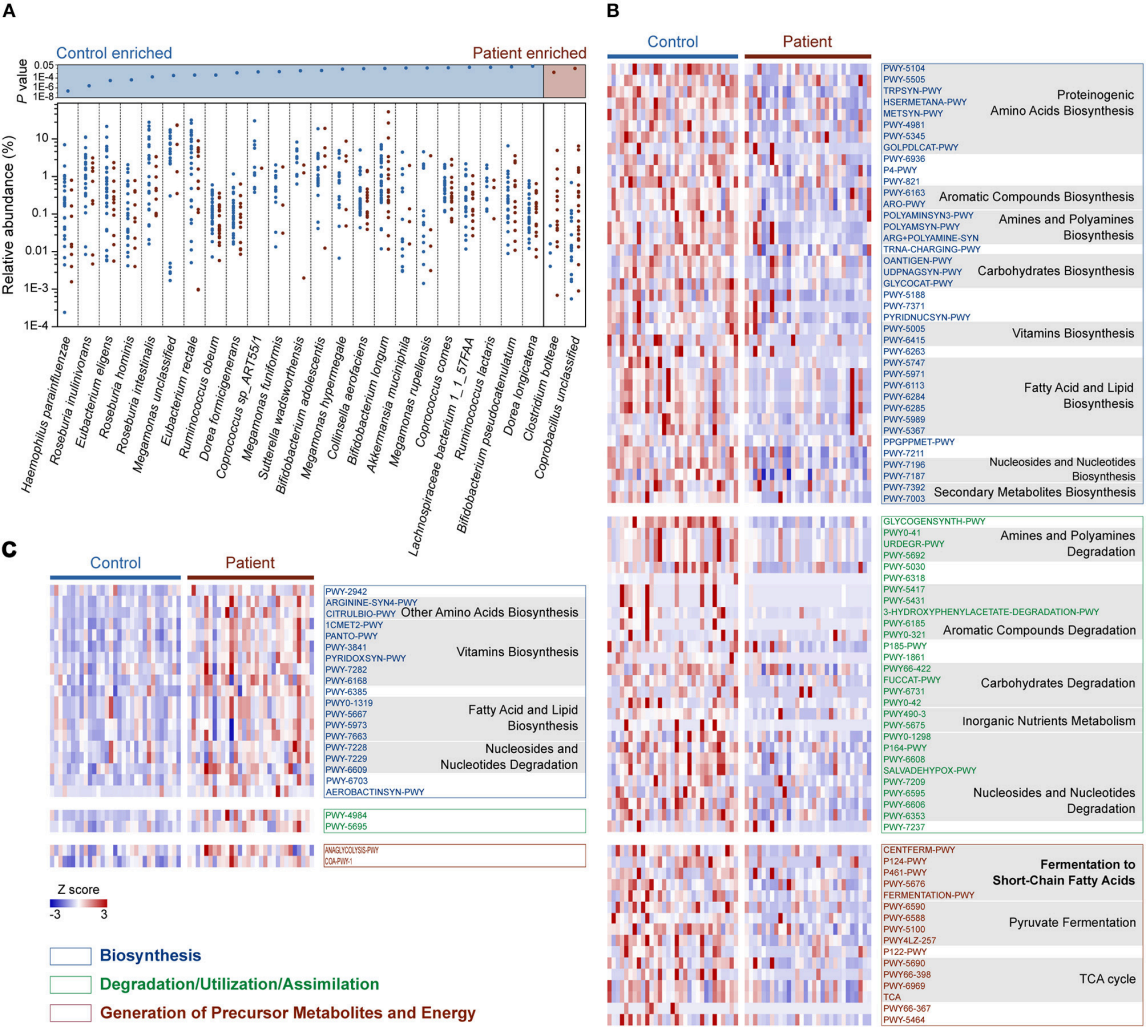
Relative abundances of differentially enriched species and metabolic pathways in the gut microbiota of the C and P groups. **(A)** Differentially abundant species between the C and P groups. *P*-values for all differentially abundant species between groups are plotted above (Wilcoxon rank-sum test, FDR < 0.1, respectively). Species (present in at least 10% samples) with mean relative abundances of more than 0.001 were considered. **(B)** Heat map showing the control-enriched metabolic pathways and **(C)** the patient-enriched metabolic pathways. Among 110 differentially abundant pathways in the C and P groups (Wilcoxon rank sum test, FDR < 0.1), 106 belonging to Biosynthesis, Degradation/Utilization/Assimilation and Generation of Precursor Metabolites and Energy are shown. The pathways are ordered by consensus functional classification. The abundance of each pathway was converted to row Z scores.

We then compared the relative abundance of each identified species across the two groups to identify the bacteria in the human gut microbiota that are altered in response to *M. tuberculosis* infection. We found that 25 species were differentially enriched in the two groups. Among these species, 23 were enriched in healthy controls, while two were more abundant in patients (Wilcoxon rank-sum test, FDR <0.1; Figure 2A and Table S5). Strikingly, nine out of the 23 control-enriched bacteria were widely reported SCFA-producing bacteria, including five butyrate producers (*Roseburia inulinivorans, R. hominis, R. intestinalis, Eubacterium rectale*, and *Coprococcus comes*), two lactate and acetate producers (*Bifidobacterium adolescentis* and *B. longum*), and two acetate and propionate producers (*Ruminococcus obeum* and *Akkermansia muciniphila*). Only two species were found enriched in the patients, including an unclassified *Coprobacillus* bacterium and *Clostridium bolteae*, the latter of which is a frequently reported autism-associated bacterium (Pequegnat et al., 2013) that has also been found to be enriched in T2D patients (Qin et al., 2012). Interestingly, both autism and T2D are closely connected with immune system abnormalities (Goines and Van de Water, 2010; Brooks-Worrell et al., 2012), and *M. tuberculosis* infections demonstrate strong interactions with the human immune systems. A causal link between mycobacterial infection and autism has been proposed (Dow, 2011), while an increased risk for *M. tuberculosis* infection in T2D patients has been recognized for centuries (Dooley and Chaisson, 2009). Therefore, further investigation is required to determine whether there is a shared alteration in immune signals that induces the similarly observed enrichment of *C*. *bolteae*, or *vice versa*, with respect to *M. tuberculosis* infection, autism and T2D.

### Functional Profiling of the Microbiome in TB Patients

In total, 110 metabolic pathways were found differentially enriched between the C and P groups (Wilcoxon rank-sum test, FDR <0.1; Table S6), among which 87 were over-represented in the healthy controls (Figure 2B), while only 23 were enriched in patients (Figure 2C), suggesting an overall decrease in metabolic potentials in TB patients. One hundred and six of the 110 differentially enriched pathways belonged to three metabolic categories: Biosynthesis, Degradation/Utilization/Assimilation, and Generation of Precursor Metabolites and Energy (Figures 2B,C; Table S6). We identified 39 pathways associated with biosynthesis that were enriched in the gut microbiota in the healthy controls. In contrast, 19 biosynthesis pathways were observed to be more abundant in TB patients. Interestingly, however, six vitamin biosynthesis pathways related to folate, pantothenate, vitamin B6, thiamine and flavin biosynthesis were highly represented in TB patients, while only the biotin and ascorbate biosynthetic pathways were more abundant in healthy controls. In addition to biosynthesis, the degradation/utilization/assimilation capacities for different types of substrates and the production of precursor metabolites and energy were decreased in patients, as reflected in the significantly lower abundance of 28 and 16 related pathways, respectively. Notably, five pathways related to the SCFA fermentation were strikingly decreased in TB patients. This result coincided with our aforementioned findings, in which SCFA-producing bacteria were significantly less abundant in TB patients.

SCFAs exert remarkable effects on host inflammatory and immune responses (Koh et al., 2016). The decreased abundance of SCFA-producers has also been observed in the gut microbiota in patients with inflammation-associated diseases, such as inflammatory bowel disease (IBD) (Kostic et al., 2014), colorectal cancer (Weir et al., 2013), and T2D (Qin et al., 2012). The loss of SCFA producers and associated pathways in TB patients may indicate elevated systemic inflammation and impairment of the systemic immune response. However, increased SCFAs in the lungs of HIV patients after antiretroviral therapy has been suggested to increase TB risk by suppressing IFN-γ and IL-17A production (Segal et al., 2017). Therefore, the role of SCFAs and the causality between active TB and the decrease of SCFA-producing bacteria should be further defined.

### Gut Bacteria Discriminate Active TB Patients From Healthy Controls

**Figure 3:**
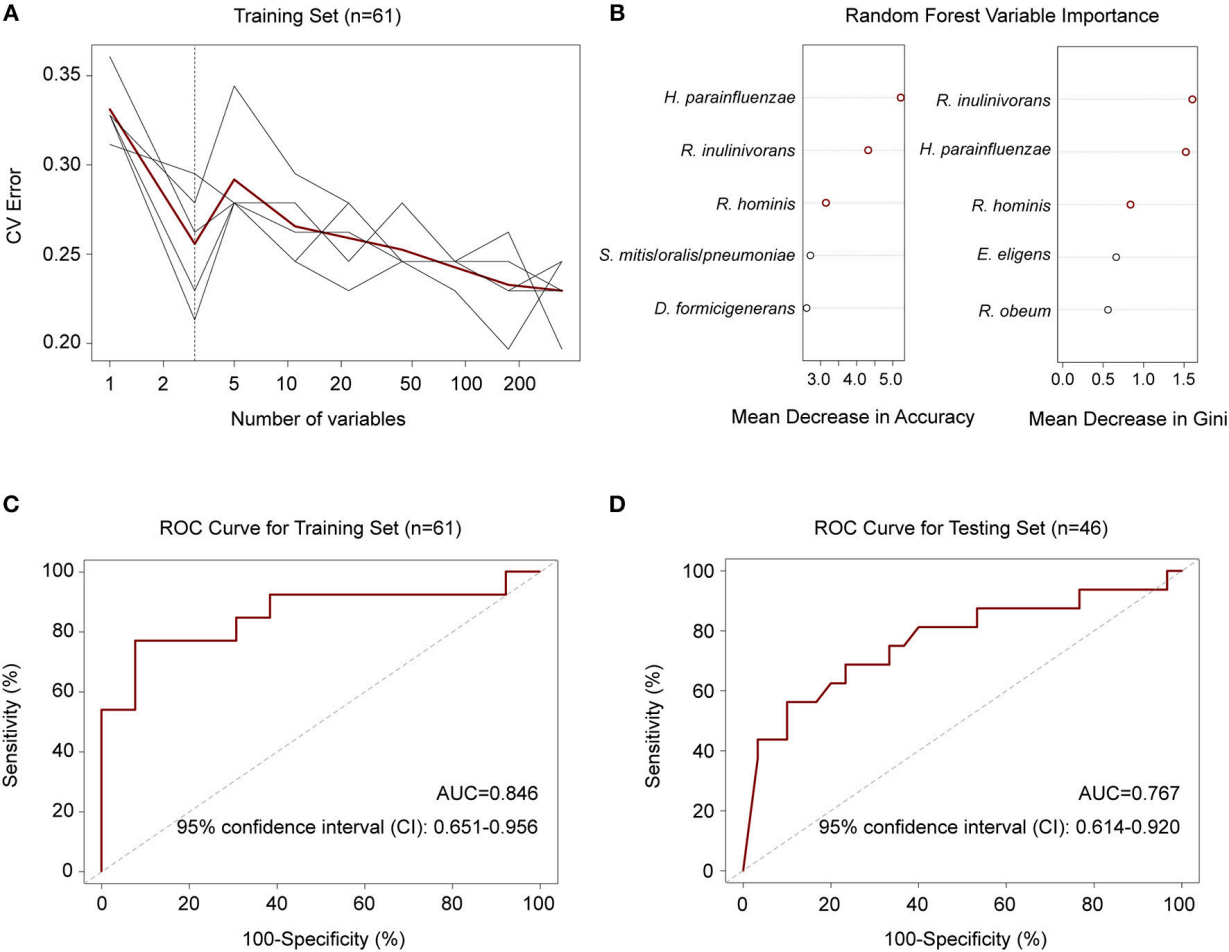
Random forest model to discriminate TB patients from healthy controls. **(A)** Five repeats of 10-fold cross-validation error. Relative abundances of 348 species in controls and patients (*n* = 31 and 30) were used to train the model. Each gray line indicates a repeat, and the red line indicated the average. The dashed line indicates the number of species in the optimal set, which was determined to be 3 species. **(B)** Random forest Mean Decrease in Accuracy and Gini. Red circles indicate the 3 species in the optimal set according to cross-validation in **(A)**. **(C)** ROC for the training set. AUC = 0.846 and the 95% CI is 0.651–0.956 (controls, *n* = 31; patients, *n* = 30). **(D)** ROC for the testing set. AUC = 0.767 and the 95% CI is 0.614–0.920 (controls, *n* = 30; patients, *n* = 16).

To explore whether there were features of the gut microbiota that discriminated *M. tuberculosis* infection status, we first employed a random forest model to distinguish between healthy and diseased states based on species abundance profiles. Five repeats of 10-fold cross-validation using the training set (*n* = 31 and 30 for controls and patients, respectively) led to the optimal selection of three species markers, *Haemophilus parainfluenzae* and two butyrate-producing bacteria, *R*. *inulinivorans* and *R*. *hominis*, for correct classification (Figures 3A,B). The performance of the model using these three species for discriminating, as assessed by the ROC analysis, achieved an AUC of 84.6%, and a 95% confidence interval (CI) of 0.651–0.956 (Figure 3C). The model also performed well when using an independent test set consisting of 16 patients and 30 healthy controls (Table S7), with an AUC of 0.767 and a 95% CI of 0.614–0.920 (Figure 3D).

Next, we performed metagenome-wide SNP calling to identify group-specific strains or SNPs. After filtering (see Methods), six species, *Bacteroides coprocola, B. stercoris, B. uniformis, B. vulgatus, Phascolarctobacterium* sp. and *Prevotella copri*, were selected for a further SNP distribution analysis (Table S8). We first compared the SNP density distributions of these six species, but no differences were observed between the groups (one-way ANOVA; Figure S1). In addition, samples belonging to different groups did not separately cluster in phylogenetic trees that were constructed based on the concatenated core genome SNPs (Figure S2).

**Figure 4:**
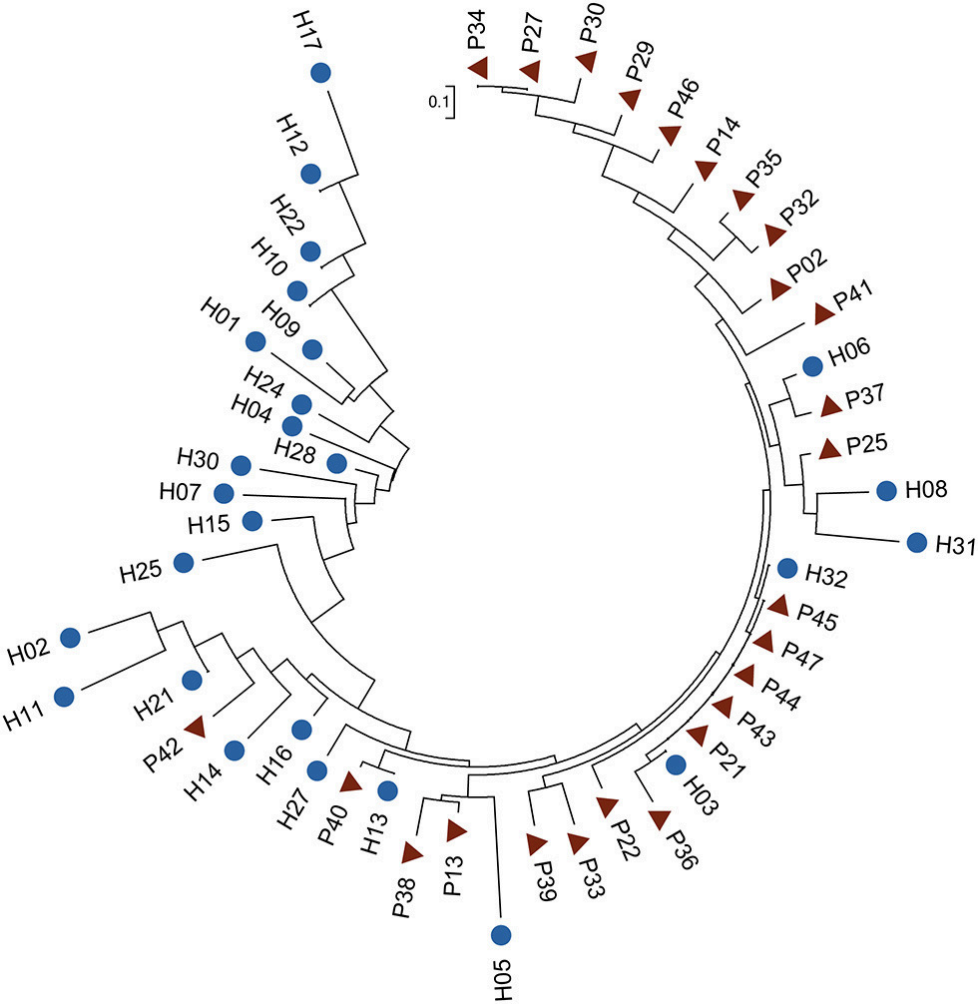
Phylogenetic tree of *B. vulgatus* based on differentially distributed core-genome SNPs. Fisher's exact test was performed to test the differences in SNP distribution (*P* < 0.01). The ML tree was constructed in MEGA 6 with 1,000 bootstrap replicates. Blue dots and red triangles indicate controls and patients, respectively.

Among the six species, *B. vulgatus* was more prevalent in the two groups, presenting in 70 samples, compared with the other five species (Table S8). We therefore examined the SNP distributions in the protein-coding regions for this species. We observed that 46 SNPs in *B. vulgatus* were differentially distributed between the two groups (Table S9). Phylogenetic analysis of these differentially distributed SNPs revealed that the control samples could be largely clustered together and were separated from the P samples, suggesting the presence of distinct mutation patterns in the species between the different groups (Figure 4). The phylogenetic analysis also indicated that the SNPs in this species were more divergent in healthy controls than those in the patients, as reflected by the differences in tree branch length.

We then determined in which genes these differentially distributed SNPs were located in. Interestingly, they were harbored within more genes associated with carbohydrate metabolism: eight SNPs were located in seven carbohydrate metabolism genes including those encoding β-galactosidase, α-N-arabinofuranosidase, α-1, 2-mannosidase, rhamnulokinase, Rhamnogalacturonides degradation protein, β-galactosidase and D-lactate dehydrogenase (Table S9). These results may suggest an altered carbohydrate preference and a resulting different carbohydrate metabolism patterns in the gut bacteria of TB patients. We should mention that our metagenomic sequencing data cannot ensure enough coverage and sequencing depth for the SNP calling of all bacterial species identified in our samples. In addition to the results we presented here, variations in other species and bacterial genes still need to be identified by using new sequencing strategies such as MetaSort (Ji et al., 2017).

## Conclusion

We applied the metagenomic sequencing approach to characterize the features of gut microbiota in TB patients. We demonstrated that the gut microbiota as well as its metabolic functions were significantly altered in TB patients. The gut bacterial species-based classifier or the strain-level SNPs have the ability to discriminate healthy from TB patients. Though we found a significant dysbiosis of the gut microbiota in TB patients, the causal relationship still needs to be determined. It is likely that these changes were caused by *M. tuberculosis* infection based on the similar results observed in murine model after *M. tuberculosis* infection (Winglee et al., 2014). However, the possibility that the dysbiosis of gut microbiota precedes and contributes to the *M. tuberculosis* infection, as suggested previously (Khan et al., 2016), cannot be excluded. Moreover, we should mention that, as only TB patients and healthy controls were included in this study, we are not clear if the gut bacteria and the suggested model have the ability to discriminate TB patients from other non TB-patients. Also, to be a potential diagnostic method for TB in the future, the accuracy and specificity of the gut microbiota-based strategy should be further validated in large-scale sampling studies. Finally, the findings we presented here were observed in patients with active TB infection; the gut microbiota signature in latent TB infection still needs to be investigated.

## Data Availability

The metagenomic sequencing data have been deposited to NCBI Sequence Read Archive database under the accession number SRP118759.

## Ethics Statement

This study was carried out in accordance with the recommendations of local ethics committee (Beijing Chest Hospital, Capital Medical University, China) with written informed consent from all subjects. All subjects gave written informed consent in accordance with the Declaration of Helsinki. The protocol was approved by the ethics committee of Beijing Chest Hospital, Capital Medical University, China.

## Author Contributions

ZS, YoH, BZ, and YX designed and supervised the project. YoH and YF performed statistical analyses. FL, YaH, and SL performed bioinformatics. JW and ZZ collected clinical samples and extracted community DNA. BL, JL, and NL constructed high through-put sequencing libraries. YoH and YF interpreted the data, and YoH wrote the manuscript. ZS and BZ read and revised the manuscript.

### Conflict of Interest Statement

The authors declare that the research was conducted in the absence of any commercial or financial relationships that could be construed as a potential conflict of interest.
