# Supplementary material for: The Gut Microbiome Signatures Discriminate Healthy From Pulmonary Tuberculosis Patients
Source: Front Cell Infect Microbiol. 2019 Apr 3;9:90. doi: 10.3389/fcimb.2019.00090 (PMC6456665; doi:10.3389/fcimb.2019.00090)
Supplement: Supplementary file 1 [file Data_Sheet_1.docx]

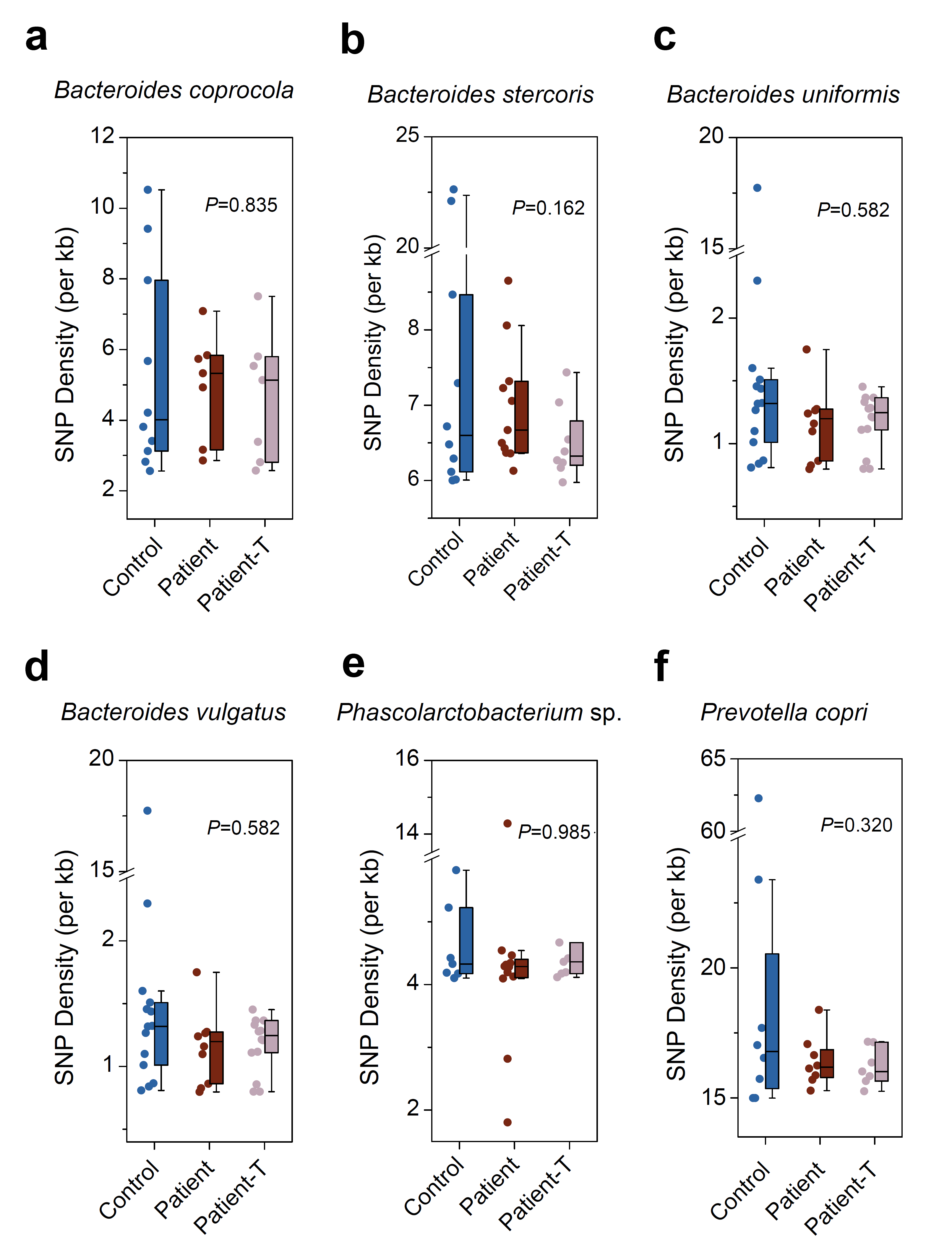


**Figure S1** SNP densities of six bacterial species. SNPs were called using the Metagenomic Intra-species Diversity Analysis System (MIDAS). SNP densities were calculated by dividing the SNP numbers with covered genome length. One-way ANOVA was performed for SNP density comparison.


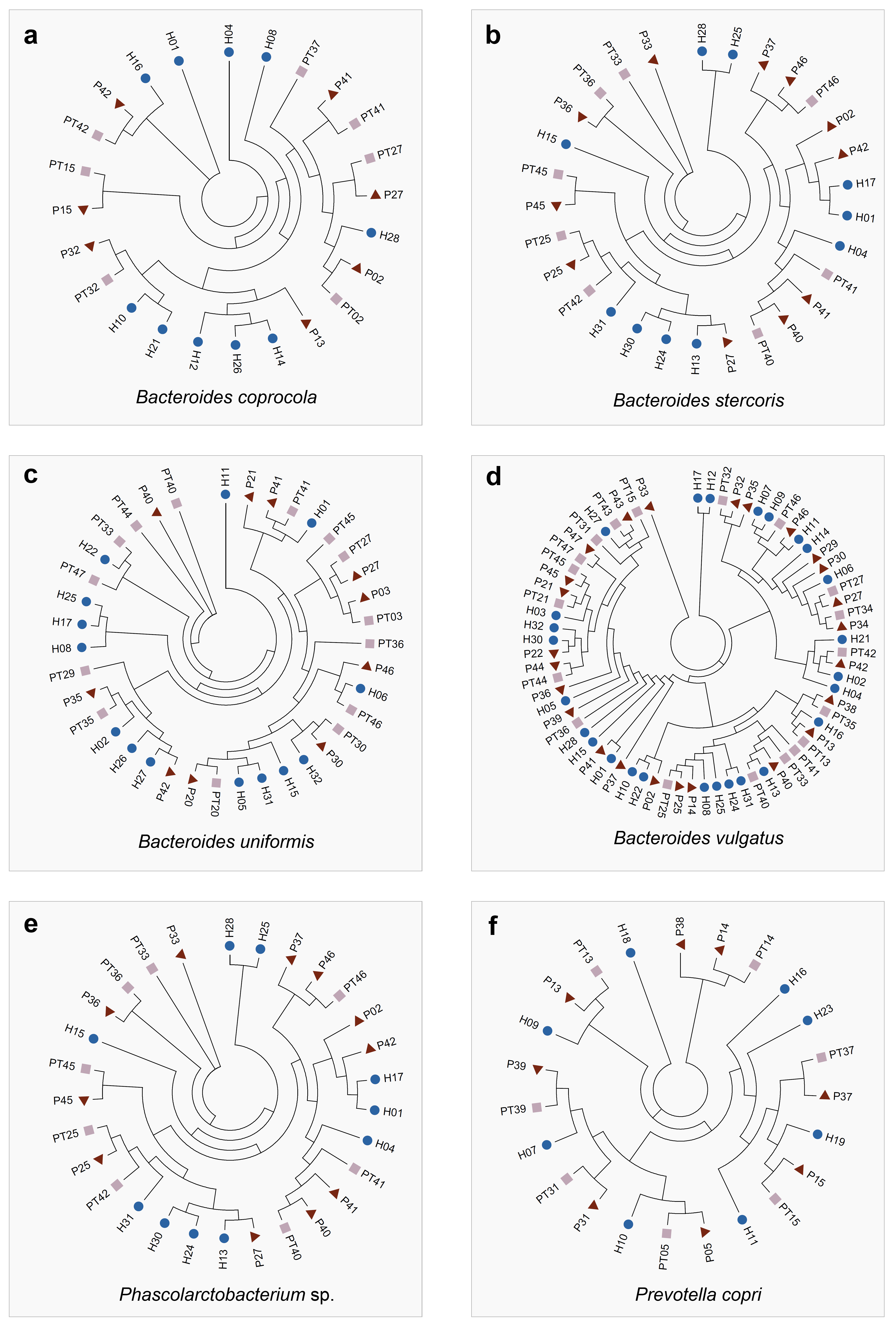


**Figure S2** Phylogenetic tree based on core-genome SNPs. Core-genome SNPs for each of the six species were concatenated for phylogenetic tree constructing. The Maximum Likelihood (ML) tree was constructed by MEGA 6 with 1000 boostrap replicates. Blue dots, red triangles and purple squares indicate control, patient and patient-T, respectively.
